# Re‐evaluation of missense variant classifications in *NF2*

**DOI:** 10.1002/humu.24370

**Published:** 2022-04-02

**Authors:** Katherine V. Sadler, Charlie F. Rowlands, Philip T. Smith, Claire L. Hartley, Naomi L. Bowers, Nicola Y. Roberts, Jade L. Harris, Andrew J. Wallace, D. Gareth Evans, Ludwine M. Messiaen, Miriam J. Smith

**Affiliations:** ^1^ Manchester Centre for Genomic Medicine, St Mary's Hospital Manchester Academic Health Sciences Centre (MAHSC) Manchester UK; ^2^ Division of Evolution, Infection and Genomics, School of Biological Sciences, Faculty of Biology, Medicine and Health University of Manchester Manchester UK; ^3^ Department of Genetics University of Alabama at Birmingham Birmingham Alabama USA

**Keywords:** classification guidelines, missense, neurofibromatosis type 2, NF2, variant classification

## Abstract

Missense variants in the *NF2* gene result in variable NF2 disease presentation. Clinical classification of missense variants often represents a challenge, due to lack of evidence for pathogenicity and function. This study provides a summary of *NF2* missense variants, with variant classifications based on currently available evidence. *NF2* missense variants were collated from pathology‐associated databases and existing literature. Association for Clinical Genomic Sciences Best Practice Guidelines (2020) were followed in the application of evidence for variant interpretation and classification. The majority of *NF2* missense variants remain classified as variants of uncertain significance. However, *NF2* missense variants identified in gnomAD occurred at a consistent rate across the gene, while variants compiled from pathology‐associated databases displayed differing rates of variation by exon of *NF2*. The highest rate of NF2 disease‐associated variants was observed in exon 7, while lower rates were observed toward the C‐terminus of the NF2 protein, merlin. Further phenotypic information associated with variants, alongside variant‐specific functional analysis, is necessary for more definitive variant interpretation. Our data identified differences in frequency of *NF2* missense variants by exon between gnomAD population data and NF2 disease‐associated variants, suggesting a potential genotype‐phenotype correlation; further work is necessary to substantiate this.

## Graphical abstract

Pathogenic/Likely Pathogenic NF2‐associated missense variants.

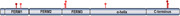

## 1 INTRODUCTION

Neurofibromatosis type 2 (NF2; MIM# 101000) is an autosomal dominant tumor predisposition condition, resulting from disruption of the *NF2* gene. Located on chromosome 22q12, *NF2* encodes the active tumor suppressor protein merlin (Trofatter et al., 1993). NF2 predisposes individuals to schwannoma development, with bilateral vestibular schwannomas (VS) being a characteristic feature (Evans et al., 1992). NF2 patients frequently experience hearing loss and tinnitus as a result of VS growth; patients may also develop neuropathies, cutaneous features, cataracts, and schwannomas on other nerves, as well as meningiomas and ependymomas (Asthagiri et al., 2009). NF2 birth incidence has been recently estimated as 1 in 28,000 (Evans et al., 2018).

The majority of pathogenic variants identified in *NF2* result in truncation of the protein product, often causing loss of protein expression or creating nonfunctional proteins (Evans, 2009). Genotype‐phenotype correlations have been observed in NF2, where protein‐truncating variants, such as frameshift or nonsense, result in more severe disease presentation than missense variants (Ruttledge et al., 1996; Smith et al., 2011). In cases where truncating variants result in a severe phenotype, a dominant negative action of the variant protein has been proposed (Evans, 2015). Variants in regulatory elements, such as splice sites and larger structural variants for example, ring chromosome 22, often result in variable disease presentation (Evans, 2009). Still, splice site variants positioned earlier in the *NF2* transcript have been associated with more severe disease presentation (Baser et al., 2005; Kluwe et al., 1998). Investigation of missense variant genotype‐phenotype correlations presents a unique challenge, as a function of an amino acid residue is not necessarily related to its position within a transcript, but rather its location within protein tertiary structures (Suckow et al., 1996).

Missense variants often represent clinical dilemmas for diagnostic services due to challenges of obtaining evidence for pathogenicity and function. Diagnostic classification of missense variants largely relies upon population frequency data and in silico predictive tools, as well as familial and functional data when available. Release of the American College of Medical Genetics and Genomics and the Association for Molecular Pathology (ACMG‐AMP) guidelines for variant interpretation (Richards et al., 2015) enabled more reproducible interpretation of variants by providing an evidence framework, facilitating more consistent clinical reporting. Subsequent revision of these guidelines has followed and the Association for Clinical Genomic Sciences (ACGS) Best Practice Guidelines for Variant Classification in Rare Disease 2020 is the framework now currently employed by the National Health Service (NHS) within the UK (ACGS best practice guidelines, 2020 https://www.acgs.uk.com/quality/best-practice-guidelines/#VariantGuidelines. Accessed 23 August 2021). The ACGS 2020 guidelines combine the detailed guidance of Richards et al. (2015), with clarifications and developments proposed by other research groups (Tavtigian et al., 2018). Key developments in the ACGS (2020) guidelines from the ACMG‐AMP include: defining variant‐specific, rather than gene‐specific, effects from functional studies, resolving scoring inconsistencies from combining evidence criteria, and the sub‐division of pathogenic, likely pathogenic and variant of uncertain significance (VUS) classifications. Further disease‐specific guidelines are currently in development through ClinGen and other curation networks, which incorporate additional disease‐associated features into variant classification; for example, loss of heterozygosity (LOH) and retention of a missense variant in a tumor would be informative for *NF2* variant classification. Recently proposed improvements in NF2 genetic severity scores suggest incorporation of merlin functional assays conducted in patient fibroblasts (Catasús et al., 2021), this evidence would be similarly valuable for *NF2* variant interpretation.

While missense variants only account for ~9% of diagnosed NF2 cases (Heineman et al., 2015), they represent >25% of observed *NF2* variants in gnomAD. This disparity may be attributed to tolerability of the NF2 protein to missense variation, but might also suggest reduced phenotype severity and disease penetrance in individuals who possess missense variants. This suggestion is supported by observed phenotypic variation in familial cases of NF2, such as the c.1604T>C p.(Leu535Pro) missense variant (Heineman et al., 2015). The c.1604T>C p.(Leu535Pro) variant has been found to segregate with disease in an extended NF2 family, where all affected individuals presented with VS at ages ranging between 16 and 80 years. A small number of this family developed other tumor types, namely meningiomas and an ependymoma. Meningiomas are often considered a mark of severity in NF2 disease and are employed as prognostic features for genomic counseling (Halliday et al., 2017); this inconsistent presentation of disease severity within one family epitomizes the challenge of defining the effect and function of such missense variants.

The aim of this study was to re‐evaluate and classify a comprehensive list of *NF2* missense variants from pathology‐associated databases, with further focus on variants identified in association with features of NF2 disease. Variants were classified according to ACGS 2020 guidelines, collating clinical and functional information where available; the intention being to provide a robust summary of current evidence that supports or refutes pathogenicity of these variants.

## 2 MATERIALS AND METHODS

### 2.1 Systematic compilation of missense variants

**Figure 1:**
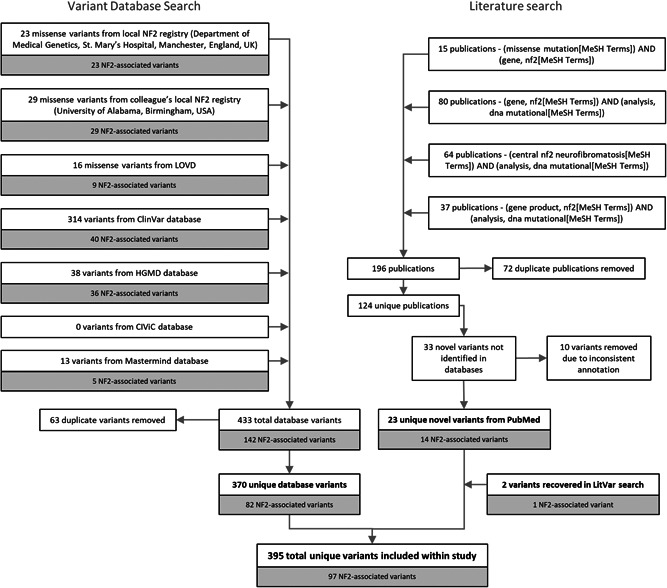
Flowchart outlining variant compilation

Compilation of known *NF2* missense variants from human disease databases was conducted systematically, primarily by clinical and public database searches, followed by literature searches for published variants. Clinical database information was obtained from NF2 registries located in the Manchester Centre for Genomic Medicine, St. Mary's Hospital, Manchester, England, UK and The University of Alabama at Birmingham, AL, USA. The publically accessible variant databases included were Leiden Open Variation Database (LOVD) (www.lovd.nl) (Fokkema et al., 2021), ClinVar NCBI (www.ncbi.nlm.nih.gov/clinvar) (Landrum et al., 2018), the Human Gene Mutation Database (HGMD) (www.hgmd.cf.ac.uk/ac/all.php) (Stenson et al., 2020), Clinical Interpretation of Variants in Cancer (CIViC) (https://civicdb.org/home) and Mastermind Genomic Search Engine (https://www.genomenon.com/mastermind). Details of duplicate variants were merged to retain relevant clinical information. A literature search was conducted through PubMed using combinations of the following MeSH terms: missense mutation, *NF2* gene, *NF2* gene product, DNA mutational analysis, central NF2/neurofibromatosis. A total of 124 unique publications were searched for novel variants. Figure 1 shows a flow chart detailing the order of variant compilation and numbers of variants included and excluded at each step. An extra literature mining step was conducted using LitVar to capture any missing variants (Allot et al., 2018). A total of 395 unique variants were included within the study.

A subset of variants identified in patients with a confirmed Manchester Criteria NF2 diagnosis (Table S1) or known NF2‐associated features, for example, unilateral VS, meningioma, ependymoma, were grouped for further analysis. A total of 97 NF2 disease‐associated variants were included, 69 of these variants appear in public databases, 17 were identifiable in the literature, the remaining 11 were exclusive to local databases and have since been submitted to public variant databases (Figure 1).

Variants outside the exonic regions of the primary *NF2* transcript RefSeq NM_000268.4 (isoform 1) were excluded from analysis, as well as variants described as nonsense, frameshifts, insertions, deletions, indels, and synonymous.

### 2.2 Variant classification tools

Evidence for clinical classification of variants was obtained and interpreted following the ACGS best practice guidelines (2020). Classification scores and posterior probabilities were also calculated for each variant (Tavtigian et al., 2018, 2020). See Table 1 for a summary of both the ACMG‐AMP (2015) and revised ACGS (2020) variant classification frameworks.

**Table 1:** A summary comparison of the ACMG‐AMP and ACGS variant classification guidelines, with additional scoring suggestions made by Tavtigian et al. (2018) and Tavtigian et al. (2020)

ACMG‐AMP classification	ACMG‐AMP evidence^a^	ACGS posterior probability threshold^b^^,^ c	Points^d^	ACGS classification		ACGS evidence
Pathogenic	1 Very strong *AND* ≥1 Strong *OR* ≥2 Moderate *OR* 1 Moderate + 1 Supporting *OR* ≥2 Supporting	*p* > 0.99	>10	Pathogenic	a	1 Very strong *AND*
						≥1 Strong *OR* ≥1 Moderate *OR* ≥2 Supporting
	≥2 Strong^e^				b	≥3 Strong
	1 Strong *AND* ≥3 Moderate *OR* 2 Moderate *AND* ≥2 Supporting *OR* 1 Moderate *AND* ≥4 Supporting				c	2 Strong *AND*
						≥1 Moderate *OR* ≥2 Supporting
					d	1 Strong *AND*
						≥3 Moderate *OR* ≥2 Moderate *AND* ≥2 Supporting *OR* ≥1 Moderate *AND* ≥4 Supporting
Likely pathogenic	1 Very strong *AND* 1 Moderate^e^	0.99 ≥ *p* > 0.90	6–9	Likely pathogenic	a	≥2 Strong
	1 Strong *AND* 1–2 Moderate					
	1 Strong *AND* ≥2 Supporting				b	1 Strong *AND*
						1–2 Moderate *OR* ≥2 Supporting
	≥3 Moderate					
	2 Moderate *AND* ≥2 Supporting				c	≥3 Moderate *OR*
						2 Moderate *AND* ≥2 Supporting *OR*
	1 Moderate *AND* ≥4 Supporting					
						1 Moderate *AND* ≥4 Supporting
Uncertain significance		0.812 ≤ *p* < 0.90	5	VUS	Hot	1 Strong + 1 Supporting *OR* 2 Moderate + 1 Supporting *OR* 1 Moderate + 3 Supporting
		0.675 ≤ *p* < 0.812	4		Warm	1 Strong *OR* 2 Moderate *OR* 1 Moderate + 2 Supporting *OR* 4 Supporting
		0.50 ≤ *p* < 0.675	3		Tepid	1 Moderate + 1 Supporting *OR* 3 Supporting
		0.325 ≤ *p* < 0.50	2		Cool	1 Moderate *OR* 2 Supporting
		0.188 ≤ *p* < 0.325	1		Cold	1 Supporting
		0.10 ≤ *p* < 0.188	0		Ice cold	
Likely benign	1 Strong *AND* 1 Supporting	0.001 ≤ *p *< 0.10	−1 to	Likely		
			−6	benign		
	≥2 Supporting					
Benign	1 Stand‐alone	*p* < 0001	<−6	Benign		
	≥2 Strong					

Abbreviations: ACGS, Association for Clinical Genomic Sciences; ACMG‐AMP, American College of Medical Genetics and Genomics and the Association for Molecular Pathology.

a Richards et al. (2015, Table 5).

b Tavtigian et al. (2018, Table 1).

c ACGS best practice guidelines (2020, Table 3 and Figure 6).

d Tavtigian et al. (2020, Table 2).

e Inconsistent evidence weighting, identified in Tavtigian et al. (2018) and resolved in ACGS (2020) guidelines.

The *NF2* transcript RefSeq NM_000268.4 was used for all in silico tool use. Variants were imported into the clinical prediction software Alamut Visual version 2.15 (SOPHiA GENETICS), in which multiple variant database information and in silico tools are embedded. Results from the following tools were exported from Alamut and factored into classification analysis: Align‐GVGD (Mathe et al., 2006), SIFT (Kumar et al., 2009), PolyPhen‐2 (Adzhubei et al., 2010), MutationTaster2 (Schwarz et al., 2014), SpliceSiteFinder‐like tool (Zhang, 1998), MaxEntScan (Yeo & Burge, 2004). Variant frequencies and ExAC constraint metrics were obtained from gnomAD v2.1.1 (gnomad.broadinstitute.org) (Lek et al., 2016).

UCSC LiftOver tool was used for any genomic co‐ordinate conversions between genome builds GRCh37/hg19 and GRCh38/hg38 (genome.ucsc.edu/cgi-bin/hgLiftOver) (Kent et al., 2002).

### 2.3 Population and frequency data

Maximum credible population allele frequency was determined using the alleleFrequencyApp (cardiodb.org/allelefrequencyapp) (Whiffin et al., 2017), and was calculated to be 1.88e‐07 for NF2, based on the following input parameters: monoallelic inheritance, disease incidence of 1 in 28,000 (Evans et al., 2018), allelic heterogeneity 0.01 and penetrance 0.95, accounting for the known rate of recurrent pathogenic variants and late disease onset. Strong benign evidence (BS1) was applied to any variants with an allele frequency equal to or higher than NF2 disease incidence (1/28,000). With a low maximum credible population allele frequency calculated (1.88e‐07), moderate pathogenicity evidence (PM2) based on frequency data was not applied to any variant observed in gnomAD as frequency values of observed variants exceeded this value.

### 2.4 Functional data

With a predicted missense constraint Z score of 2.29 in ExAC, *NF2* is considered moderately intolerant of variation. However, only Z scores ≥3.09 are considered significant within the ACGS guidelines and therefore variants in *NF2* are ineligible for application of evidence for missense constraint (PP2).

The DECIPHER database (Firth et al., 2009) was used to investigate possible mutational hotspots or identify regional constraint within functional domains of the NF2 protein. However, no specific structural regions displayed significant association with missense constraint. Therefore, ACGS evidence of mutational hotspots and functional domains without benign variation (PM1) was not applied to any of the variants in this study.

While functional work has been conducted and published on a number of variants included within this study, evidence from functional studies (PS3) was only applied to five specific variants as repeated and rigorous publications describing variant‐specific effects on protein function were available for them. No functional data from RNA analysis was available for variants predicted to impact splicing.

### 2.5 Computational data

Multiple in silico tools were used for variant effect prediction; meta‐predictor REVEL (Rare Exome Variant Ensemble Learner) was used as the deciding score for evidence use (PP3 and BP4) if other tools were in conflict (Ioannidis et al., 2016), as it is one of the best performing meta‐predictors (Wilcox et al., 2021). REVEL scores ≥0.7 were considered pathogenic and ≤0.4 benign. ClinPred (Alirezaie et al., 2018) meta‐predictor scores were also produced for variants, although were not included as evidence for ACGS variant classification.

Splice prediction tools were also interpreted and applied as evidence, as suggested in the ACGS 2020 guidelines. Variants that received MaxEntScan (Yeo & Burge, 2004) predictions of >15% score reduction compared to reference allele, and SpliceSiteFinder‐Like (Zhang, 1998) predictions with >5% reduction, had PP3 computational evidence of pathogenicity applied in their classification.

### 2.6 Clinical information

If phenotype was described, patients who fitted Manchester Criteria for NF2 disease (Table S1) (Evans et al., 1992; Smith et al., 2017) were considered to have phenotypic specificity for a disease of single etiology (PP4), applied as supporting evidence of pathogenicity. Where possible, family history and segregation data was applied to the evidence framework.

### 2.7 Other databases

COSMIC (www.cancer.sanger.ac.uk) (Tate et al., 2019) was used in the investigation of variants that were observed in somatic samples. CanVar‐UK cancer predisposition gene variant database (www.canvaruk.org) was used in the search for further variant information, as well as links to structured search engine requests.

## 3 RESULTS

### 3.1 Summary of variant classifications

From the 395 total variants interpreted in this study, 375 were classified as VUS. The majority of VUSs (73%) were identified exclusively from ClinVar without accompanying phenotypic information, these variants were observed in large‐scale classification studies without focus on NF2 disease (Nykamp et al., 2017). Variants, shown in Table 2, were placed into further VUS temperature categories in line with ACGS recommendations (Table 1). A complete list of variants and the evidence categories applied to their classification can be found in Table S2.

**Table 2:** Summary of variant classifications of all missense variants identified in *NF2* from pathology databases, with further grouping into NF2 disease‐associated *NF2* variants

Classifications	Variants in *NF2*	
	All database variants	NF2 disease‐associated
Benign	0	0
Likely benign	12	6
VUS (ice cold)	85	14
VUS (cold)	87	10
VUS (cool)	96	16
VUS (tepid)	83	21
VUS (warm)	17	16
VUS (hot)	7	6
Likely pathogenic	6	6
Pathogenic	2	2
Total	395	97

Abbreviation: VUS, variant of uncertain significance.

While 395 variants were collated in total, only 97 were identified in cases with confirmed NF2‐associated phenotypic features (Table 2). All variants classified as likely pathogenic and pathogenic were identified in association with NF2 disease presentation, and were therefore assigned to both data groups in Table 2.

Seventeen *NF2* missense variants had in silico computational evidence of pathogenicity (PP3) applied by splicing prediction tool scores, MaxEntScan (Yeo & Burge, 2004) and SpliceSiteFinder‐Like (Zhang, 1998), in the absence of a pathogenic REVEL metascore. All seventeen of these potential splicing variants remain classified as VUS.

Interestingly, one variant, c.1532A>G, predicted to produce the missense change, p.(Asp511Gly), and not predicted to affect splicing by the MaxEntScan and SpliceSiteFinder‐Like tools, was shown to affect splicing by RNA analysis (methods described in Piotrowski et al., 2014). This variant results in an out of frame mis‐spliced transcript, r.1533_1575del, p.(Asp511Valfs*24). Confirmation of aberrant splicing allowed application of strong evidence for pathogenicity from in vitro studies (PS3), resulting in a likely pathogenic classification.

### 3.2 Conflict with existing classifications

When all variant classifications were compared to existing ClinVar interpretations, 17 variants were in conflict with current submissions, seen in Table 3. The vast majority of these variants were downgraded in pathogenicity class.

**Table 3:** Variants with conflicting classification to existing submissions in ClinVar. Likely pathogenic (b/c) = variant subclassifications as per Table 1

Sequence change	Amino acid change	ClinVar (number of submissions)	ACGS classification	NF2 phenotype observed
c.2T>C	p.(Met1Thr)	Likely pathogenic (1)	VUS (warm)	Unknown
c.613A>G	p.(Met205Val)	VUS (3)/Benign (1)	Likely benign	Associated
c.641T>C	p.(Leu214Pro)	VUS (1)/Likely pathogenic (1)	VUS (hot)	Yes
c.658A>T	p.(Asn220Tyr)	Pathogenic (1)	Likely pathogenic (c)	Yes
c.1052G>A	p.(Arg351His)	VUS (2)	Likely benign	Associated
c.1079T>C	p.(Leu360Pro)	Pathogenic (1)	Likely pathogenic (b)	Associated
c.1385G>A	p.(Arg462His)	VUS (2)	Likely benign	Unknown
c.1387G>A	p.(Glu463Lys)	VUS (1)/Likely benign (2)	Likely benign	Unknown
c.1439C>T	p.(Thr480Met)	VUS (2)	Likely benign	Yes
c.1451T>C	p.(Met484Thr)	VUS (1)/Likely benign (1)/Benign (1)/not provided (1)	Likely benign	Unknown
c.1540A>G	p.(Met514Val)	VUS (4)/benign (1)	Likely benign	Yes
c.1550T>C	p.(Leu517Pro)	Pathogenic (1)	VUS (warm)	Yes
c.1613A>C	p.(Gln538Pro)	Pathogenic (1)	Likely pathogenic (b)	Yes
c.1639G>A	p.(Glu547Lys)	VUS (1)/Likely benign (2)	Likely benign	Associated
c.1701C>G	p.(Asp567Glu)	VUS (3)	Likely benign	Unknown
c.1753G>A	p.(Ala585Thr)	VUS (3)	Likely benign	Unknown
c.1774T>C	p.(Phe592Leu)	VUS (4)/Likely benign (1)	Likely benign	Unknown

*Note*: Associated—observed in individual with features associated with NF2 but without fulfilling Manchester NF2 criteria. Yes—observed in individual fulfilling Manchester NF2 criteria. *NF2* transcript RefSeq NM_000268.4.

### 3.3 Rate of variation across NF2

**Figure 2:**
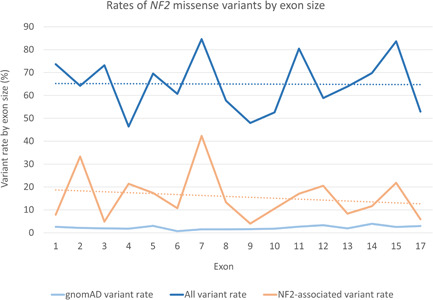
A comparison of rates of *NF2* missense variants in gnomAD v2.1.1 (controls), all variants identified within this study, and NF2 disease‐associated variants. Rates were calculated as a percentage of the number of variants in comparison to exon size in amino acids. Assumed benign variation in the gnomAD v2.1.1 (controls) data set remains consistent across the gene. In contrast, there is an increased rate of variation in a number of exons for variants identified in pathology databases

The number of variants identified in each exon of *NF2* was compared to exon size in amino acids. Missense variants recorded within gnomAD occurred at a highly consistent rate across the *NF2* transcript, Figure 2. When considering all 395 *NF2* variants identified in this study rates per exon differed, yet the average trendline remained consistent across the gene (Figure 2). Exon 4 possessed the lowest rate of variation by size and exon 7 the highest. Considering the 97 NF2‐associated variants, rates of variation changed for a number of exons but remained highest in exon 7. Approximately half of all variants in exons 2, 4, and 7 were identified in association with an NF2 phenotype. The lowest rates of NF2‐associated variants were observed in exons 3, 9, and 17. Notably, the average trendline for NF2‐associated variants decreased toward the end of the gene (Figure 2).

**Figure 3:**
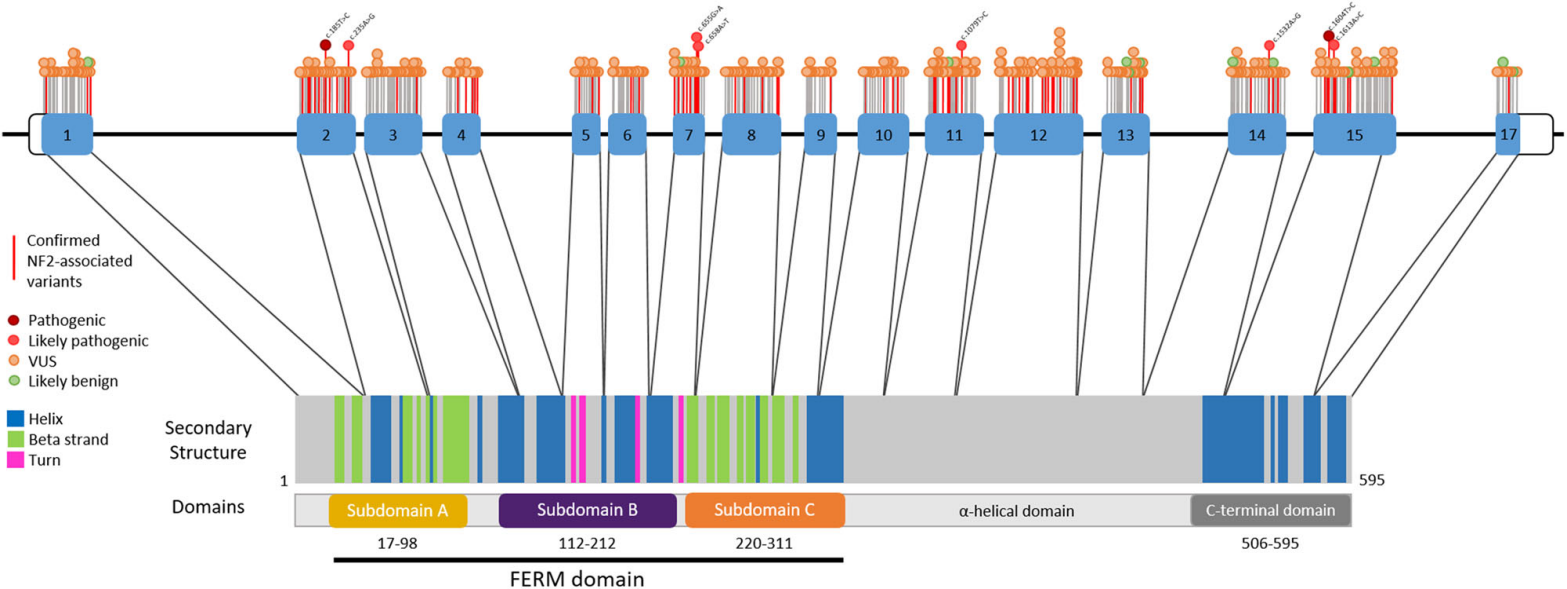
*NF2* isoform 1. Missense variants with corresponding classifications are labeled on the exon‐intron structure at the top of the figure. Confirmed NF2‐associated variants are tagged in red. Likely pathogenic and pathogenic variants are labeled with variant nomenclature. Exon boundaries are highlighted on a schematic of the translated protein product with annotated secondary structures, as well as the tertiary domains of the protein. *NF2* transcript RefSeq NM_000268.4

Identified variants were plotted across a schematic of isoform 1 of the *NF2* gene structure to highlight potential mutational hotspots (Figure 3); context of secondary and tertiary structure motifs was also included (Shimizu et al., 2002). Missense variants occur across all exons of *NF2*, yet localized clustering of NF2‐associated variants are observed in some exons, such as the 5′ region of exon 15. The high rates of NF2‐associated variants across exons 2 and 7 are observable in Figure 3.

### 3.4 Somatic variants

From the 395 variants collated within this study, 39 had been observed exclusively in somatic samples. Many of the somatic samples were obtained from schwannoma and meningioma tumors, however, 15 of the variants were identified exclusively in non‐NF2 related tumor types, such as liver, breast and lung cancers (Table S2).

## 4 DISCUSSION

The vast majority of missense variants identified within *NF2* are classified as variants of uncertain significance in accordance with the ACGS 2020 guidelines. Unfortunately, these variants remain as clinical interpetation dilemmas without sufficient evidence to ascribe or discount them as disease causing. While the VUS temperature scale provides further insight into the possible pathogenicity of a variant, many variants remain at the “cooler” end of the scale with little compelling evidence available, see Table 1 for ACGS VUS sub‐classifications. The novel temperature scaling, suggested in the ACGS 2020 guidelines, provides a system for prioritizing evidence collection for variants of uncertain significance; for example, obtaining further phenotypic information on patients possessing a specific VUS may enable upgrading of variant pathogenicity at minimal cost. Approximately one‐third of variants observed in association with NF2 phenotypic features were grouped into “warm” or “hot” VUS and pathogenic classification boundaries; this is primarily due to the clinical and familial evidence available for these variants. Clinical information was unavailable for a large proportion of the variants included within this study and therefore other institutions may be able to reclassify variants upon application of such accompanying data. Similarly, if functional data on variant‐specific effects was available, such as RNA studies on possible splicing variants, application of stronger lines of evidence (PS3) and therefore more resolute variant classification would be possible. The need for inclusion of higher performing splice prediction tools within the ACGS guidelines, alongside the utility of RNA studies is exemplified by variant c.1532A>G. While MaxEntScan and SpliceSiteFinder‐Like tool did not produce significant splice prediction scores, mRNA analysis from a patient sample confirmed aberrant splicing of *NF2*. The apparent missense variant actually results in frameshifted transcript, r.1533_1575del p.(Asp511Valfs*24), which is predicted to lead to nonsense‐mediated decay. Confirmation of aberrant splicing through functional analysis allowed application of strong evidence for pathogenicity (PS3), and upgrading of the variant classification to likely pathogenic. SpliceAI (Jaganathan et al., 2019) splice prediction scores were obtained for each of the variants included within this study, but were not employed for classification purposes following current ACGS guidelines. SpliceAI scores were considered with the following weighting >0.8 high confidence prediction, >0.5 confident prediction, 0.2–0.5 lower confidence prediction. Variant c.1532A>G received a high confidence SpliceAI score, predicting a donor gain splicing event, lending support for the inclusion of SpliceAI in variant prediction. A further 13 *NF2* missense variants with confident SpliceAI consequence predictions remain without functional evidence (PS3) in our variant list (Table S2), these variants represent promising candidates for RNA studies that may generate further evidence of pathogenicity and therefore variant reclassification.

Evidence of mutational hotspots and functional domains (PM1), was not applied to any of the variants in this study as no specific structural domains of *NF2* display missense constraint in DECIPHER, as outlined in the ACGS 2020 guidelines. However, with our observations of variant clustering in different domains of merlin, alongside a number of studies describing domain‐specific interactions of the protein function (Shimizu et al., 2002; Stokowski & Cox, 2000), it seems likely that regional constraint could be better defined for *NF2*. Identifying areas of regional constraint would enable the application of moderate evidence for pathogenicity that might enable the revision of a number of variants into likely pathogenic and pathogenic classifications. Exploring ways to redefine regional constraint and domain function for *NF2* may prove valuable in the curation of NF2 disease‐specific variant interpretation guidelines. Full details of the 395 *NF2* missense variants is available in Table S2.

From the 17 variants for which reclassification conflicted with the existing ClinVar classification, most were downgraded in pathogenicity when following ACGS recommendations. The majority of the downgraded variants from ClinVar had prior pathogenicity determined based on evidence considered weak by both the ACMG‐AMP and ACGS guidelines. For example, the c.658A>T p.(Asn220Tyr) variant is classified as pathogenic within ClinVar based on in silico analysis, segregation within a single family and a singular functional study. Yet, when this evidence is considered within the ACGS framework, the c.658A>T p.(Asn220Tyr) variant should be considered likely pathogenic, as no strong evidence is suitably applicable. Another consideration of ClinVar variant classifications is the age of the studies that were used to assign pathogenicity; a number of variants were submitted to ClinVar before the inception of the clinical variant interpretation guidelines suggested by Richards et al. (2015), and therefore evidence is often applied with inconsistent weighting in these earlier submissions.

When considering missense variant rates by exon size, a highly consistent rate of assumed benign variation was observed in the gnomADv2.1.1 (controls) data set (Figure 2). In contrast, the variants collated from pathology databases for this study demonstrated differing rates of variation by exon. The comparable pattern of variant rates between “all database variants” and “NF2‐associated variants” in Figure 2 suggests that a considerable fraction of “all database variants” are potentially pathogenic and would be associated with an NF2 phenotype if clinical features were provided. Exon 7 possessed the highest rate of variation, with approximately half of its variants occurring within a codon possessing at least one other recorded missense variant (Figure 3). Exon 7 also contained the highest rate of NF2‐associated variants. Spanning the linker region of subdomains B and C of the FERM domain in the merlin protein (Figure 3), the sequence of exon 7 in *NF2* is highly conserved across the ERM (ezrin, radixin, moesin) protein superfamily (Shimizu et al., 2002). The sequence conservation of exon 7, alongside the high rate of NF2‐associated missense variants, suggests that alteration of amino acid residues in this region may disrupt critical biophysical interactions of the merlin protein. For example, the exon 7 variant c.658A>T p.(Asn220Tyr) has been reported to display reduced binding to scaffolding protein EBP50 (Stokowski & Cox, 2000); Shimizu et al. (2002) theorized that this may be due to altered residue contacts resulting in changes to subdomain orientation.

Rates of NF2‐associated variants decreased toward the end of the *NF2* gene, which may suggest that variants positioned later in the gene transcript are less likely to disrupt function of the protein, similar to the genotype‐phenotype correlation observed in *NF2* splice variants (Baser et al., 2005; Kluwe et al., 1998). Moreover, the single NF2 disease‐associated variant identified in exon 17 was observed in a somatic astrocytoma sample from one individual. Astrocytomas are observed very rarely in association with NF2 (Gene Reviews—Neurofibromatosis 2, 2018. https://www.ncbi.nlm.nih.gov/books/NBK1201/. Accessed September 02, 2021) and it is possible that this variant was acquired somatically in the tumor and is not related to NF2 disease. As the two predominant isoforms of merlin possess variant C‐terminal ends (Shimizu et al., 2002), it is possible there is transcript redundancy that reduces the pathological effect of variants toward the end of the gene. As only isoform 1 of *NF2* has been analyzed within this study it should be considered that some variants may confer transcript‐specific effects currently unaccounted for in our interpretation.

Fifteen of the *NF2* missense variants included in this study were observed exclusively in somatic samples from non‐NF2 related tumors, and this is consistent with previous observations of somatic *NF2* variants in multiple cancer types, such as mesothelioma, liver, and large instestine cancers (Schroeder et al., 2014). Merlin is a known tumor suppressor, regulating multiple cell signaling pathways associated with cell proliferation and therefore tumorigenesis of multiple cancer types (Cui et al., 2019; Trofatter et al., 1993).

With the current absence of NF2 disease‐specific guidelines for variant classification, we propose additional presentation features that could be considered for *NF2* variant interpretation under the ACGS 2020 framework. Individuals meeting Manchester NF2 criteria with an identifiable germline *NF2* rare missense variant in the absence of other detectable variants, in addition to somatic *NF2* LOH with retention of the missense variant on the *trans*‐allele, would provide moderate evidence for pathogenicity of a missense variant. Furthermore, observed mosaicism of an identical *NF2* rare missense variant in two tumor samples, or at low frequency in blood, would be strong evidence for pathogenicity of the variant in the absence of other variant identification. An example of the utility for this suggested evidence criteria can be seen for variant c.655G>A p.(Val219Met), which has been described in somatic samples and cases of mosaic NF2 identified through multiple tumor genotyping (Heineman et al., 2015). Since missense variants generally lead to a milder phenotype, they are more likely to be seen as non‐mosaic variants (Evans et al., 2013). The frequent observation of c.655G>A p.(Val219Met) mosaicism—five mosaic NF2 patients seen in Manchester laboratory—suggests the variant may confer a severe functional effect, as low‐level mosaic patients still present with a clinical NF2 phenotype. Application of the suggested NF2 disease‐specific evidence for mosaicism would enable reclassification of this variant from likely pathogenic (c) to pathogenic (d). Both of these specific genotypic observations could be incorporated into ACGS 2020 variant interpretation guidelines by increasing the strength of the PP4 evidence class to moderate or strong, “patient phenotype or family history is highly specific for a disease with a single genetic etiology.”

In conclusion, most *NF2* missense variants remain classified as variants of uncertain significance after application of current ACGS guidelines. Our observation of differing missense variant rates by exon of *NF2*, with fewer NF2‐associated variants toward the C‐terminus of merlin, is suggestive of a potential genotype‐phenotype correlation, although further work is necessary to substantiate this. While we provide a comprehensive list of *NF2* missense variants, it is not exhaustive, and we encourage other researchers within the field to submit novel variants to public databases. This is particularly significant with the anticipation of ClinGen NF2 disease‐specific variant interpretation guidelines. There is an unmet demand for both clinical descriptions in association with reported variants, alongside functional analysis of variant‐specific effects on merlin, including RNA studies, which are necessary for more definitive variant interpretation.

## AUTHOR CONTRIBUTIONS

*Conceptualization*: Miriam J. Smith and Ludwine M. Messiaen. *Data curation*: Katherine V. Sadler, Charlie F. Rowlands, D. Gareth Evans, Ludwine M. Messiaen, and Miriam J. Smith. *Formal analysis*: Katherine V. Sadler, Philip T. Smith, Claire L. Hartley, Naomi L. Bowers, Nicola Y. Roberts, Jade L. Harris, and Andrew J. Wallace. *Funding acquisition*: D. Gareth Evans and Miriam J. Smith. *Investigation*: Katherine V. Sadler. *Visualization*: Katherine V. Sadler. *Writing—original draft*: Katherine V. Sadler. *Writing*—*review & editing*: Katherine V. Sadler, Charlie F. Rowlands, Philip T. Smith, Claire L. Hartley, Naomi L. Bowers, Nicola Y. Roberts, Jade L. Harris, Andrew J. Wallace, D. Gareth Evans, Ludwine M. Messiaen, and Miriam J. Smith.

## CONFLICTS OF INTEREST

The authors declare no conflicts of interest.

## ETHICS STATEMENT

Ethical approval for the use of anonymised samples from the Manchester Centre for Genomic Medicine archive was obtained from the North West—Greater Manchester Central Research Ethics Committee (reference 10/H1008/74). Ethical approval for the use of deidentified data from the UAB Medical Genomics Laboratory was obtained from the UAB Institutional Review Board, project number 080926009.

## Supporting information

Supporting information.

**Table S2**.

## Data Availability

The data that support the findings of this study are available on request from the corresponding author. The data are not publicly available due to privacy or ethical restrictions. ACGS best practice guidelines (2020): https://www.acgs.uk.com/quality/best-practice-guidelines/#VariantGuidelines. Align‐GVGD: http://agvgd.hci.utah.edu/. AlleleFrequencyApp: cardiodb.org/allelefrequencyapp. CanVar‐UK: www.canvaruk.org. CIViC: https://civicdb.org/home. ClinPred: https://sites.google.com/site/clinpred/. ClinVar NCBI: www.ncbi.nlm.nih.gov/clinvar. COSMIC: www.ncbi.nlm.nih.gov/clinvar. DECIPHER: https://www.deciphergenomics.org/. ExAC: https://exac.broadinstitute.org/. gnomAD v2.1.1: gnomad.broadinstitute.org. Human Gene Mutation Database: www.hgmd.cf.ac.uk/ac/all.php. Leiden Open Variation Database: www.lovd.nl. LitVar: https://www.ncbi.nlm.nih.gov/CBBresearch/Lu/Demo/LitVar/#!?query=. Mastermind Genomic Search Engine: https://www.genomenon.com/mastermind. MutationTaster2: https://www.mutationtaster.org/. PolyPhen‐2: http://genetics.bwh.harvard.edu/pph2/. REVEL: https://sites.google.com/site/revelgenomics/. UCSC LiftOver tool: genome.ucsc.edu/cgi-bin/hgLiftOve
