# Supplementary material for: Re‐evaluation of missense variant classifications in NF2
Source: Hum Mutat. 2022 Apr 2;43(5):643–54. doi: 10.1002/humu.24370 (PMC9323416; doi:10.1002/humu.24370)
Supplement: Supplementary file 1 — Supporting information. [file HUMU-43-643-s002.pdf]

**Table S1. Current and revised Manchester Criteria for neurofibromatosis type 2 (NF2)**
1. Bilateral vestibular schwannomas <70 **OR**
2. Family history* of NF2 **AND** unilateral VS <70 **OR**
3. Family history* of NF2 **OR** UVS **AND** any two of: meningioma, glioma, neurofibroma, schwannoma, cataract, cerebral calcification (if UVS + ≥2 non intradermal schwannomas need negative *LZTR1* testing) **OR**
4. Multiple meningiomas (two or more) **AND** any two of: UVS, glioma, neurofibroma, schwannoma, cerebral calcification **OR**
5. Constitutional or mosaic pathogenic *NF2* mutation in blood or identical mutations in two distinct tumours
*VS = Vestibular Schwannoma, UVS = Unilateral Vestibular Schwannoma * First degree relative*
